# Effects of daytime variation on pain intensity and analgesic requirement after cesarean section: a retrospective and prospective study

**DOI:** 10.1080/07853890.2025.2528453

**Published:** 2025-07-05

**Authors:** Bin Shu, Ai Yan, Yonggang Liang, Jianrong He, Jie Chen, Xuehan Chen, He Huang, Guangyou Duan

**Affiliations:** Department of Anesthesiology, The Second Affiliated Hospital, Chongqing Medical University, Chongqing, China

**Keywords:** Surgery, postoperative pain, numerical rating scale, morning, afternoon, endorphin

## Abstract

**Background:**

This study compared postoperative pain and analgesic requirements in women who underwent cesarean section (CS) in the morning and afternoon.

**Summary background data:**

It is unclear whether there was difference in postoperative pain intensity and analgesic requirements between morning and afternoon CS.

**Methods:**

A single-center retrospective cohort study was conducted to compare postoperative analgesic requirements between women who underwent CS during 06:00–12:00 (morning group, *n* = 104) and 12:00–18:00 (afternoon group, *n* = 104). Then, a prospective cohort study was conducted, including 55 women each in the morning and afternoon groups. The primary outcome was area under the curve (AUC) of the pain NRS during 24 h after the CS, and analgesic consumption was recorded. Pressure pain sensitivity and serum interleukin-6 and endorphin levels were detected pre- and postoperatively.

**Results:**

The frequency of analgesic requirements 24 h after CS in the afternoon group was significantly higher than morning group in the retrospective cohort. The AUC of pain NRS and analgesic consumption during 24 h after afternoon CS were significantly higher than those after morning procedures. Significantly lower pressure pain tolerance, higher postoperative serum endorphin levels and higher levels of interleukin-6 were found in the afternoon group compared to those preoperatively, but not in the morning group.

**Conclusion:**

CS performed in the afternoon predicted severer postoperative pain compared to those performed in the morning, which might be associated with lower postoperative pain tolerance and more drastic responses to surgery. This finding needs to be considered in pain treatment after CS in the future.

**Clinical trial number and registry URL:**

ChiCTR2000039720, www.chictr.org.cn.

## Introduction

In 2018, the cesarean section (CS) rate reached 36.7% in China [1] and almost one in three births in the United States involved CS [2]. An estimated 29.7 million births were performed with CS in 2015 [3], highlighting the procedure as one of the most common surgeries worldwide. Postoperative pain is an unavoidable complication of CS that can seriously affect the patient’s recovery and satisfaction while increasing the postoperative length of stay as well as direct costs [4,5]. Despite the development of numerous analgesics and strategies for managing postoperative pain, the incidence of moderate to severe pain after CS has reached approximately 50% [6–8]. Therefore, post-CS pain remains a significant issue to be addressed in clinical practice.

Identifying the factors affecting postoperative pain can help to improve pain therapies, as this will enable clinicians to explicitly target individuals at a high risk of moderate to severe postoperative pain by implementing appropriate preoperative screening methods [9–12]. Previous reports have demonstrated variations in many vital biological processes based on the time of day [13,14]. For example, a study found that the incidence of major adverse cardiac events was lower in patients undergoing cardiac surgery in the afternoon than in those undergoing surgery in the morning [15]. Other studies have highlighted the need to consider diurnal rhythmicity to improve therapeutic strategies for pathological pain [16,17]. We noted that women receiving CS in the afternoon often complained of higher pain intensity in our clinic. We thus hypothesized that daytime variation might affect post-CS pain intensity. In a large retrospective analysis over a period of ten years showed fewer caesarean sections are performed at nighttime (8.7 vs. 10.1%) [18]. Considering most of the CS are performed in the daytime, we thought it is necessary to explore the potential difference in postoperative pain intensity between morning and afternoon CS. Based on the information, we hypothesized that daytime variation can affect post-CS pain intensity and analgesia requirement. Thus, the aim of the current study was to investigate the potential difference in pain intensity between CS performed in the morning and in the afternoon using both retrospective and prospective study designs.

## Methods

### Study design

This study involved a retrospective cohort analysis and a prospective cohort trial, both of which were conducted in accordance with the Declaration of Helsinki. Data for the retrospective study were obtained from the electronic medical record and the postoperative analgesia system. The work has been reported in line with the STROCSS criteria [19]. The study was approved by the Institutional Ethics Committee of the Second Affiliated Hospital of Chongqing Medical University (approval ID: 2020-107) in Chongqing, China, on 26 October 2020. Written informed consent was obtained from all participants in the prospective study, and the Ethics Committee of the Second Affiliated Hospital of Chongqing Medical University waived informed consent for the retrospective portion of the study in accordance with the national legislation and the institutional requirements. The prospective trial was registered with the Chinese Clinical Trial Registry (www.chictr.org.cn) prior to patient enrolment (ChiCTR2000039720). The study was conducted at the Second Affiliated Hospital of Chongqing Medical University, and no changes to the methods were made after commencement of the trial. Following deidentification and signed compliance with the data access agreement, the raw study data can be acquired on request to the corresponding author via email.

### Patients

**Figure 1.**
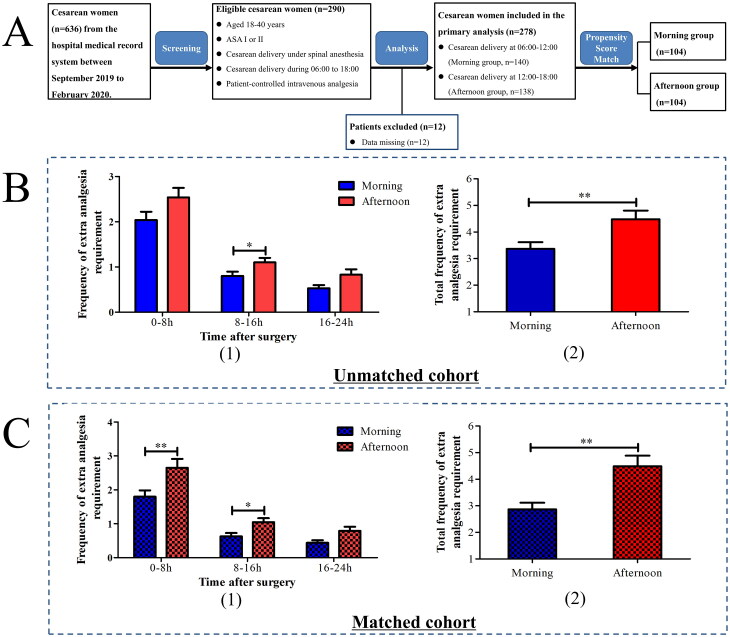
Study flowchart (A) and comparisons between the morning and afternoon groups in the primary cohort (B) and propensity score-matched cohort (C). *p < 0.05; **p < 0.01.

In the retrospective study (Figure 1A), from September 2019 to February 2020, women aged 20–40 years who underwent CS via a transverse incision under spinal anesthesia from 06:00 to 18:00, as well as with American Classification of Anesthesiologists (ASA) class I–II, singleton pregnancy, and receipt of patient-controlled intravenous analgesia (PCIA), were considered eligible. Patients with these missing data were excluded from the retrospective analysis.

**Figure 2.**
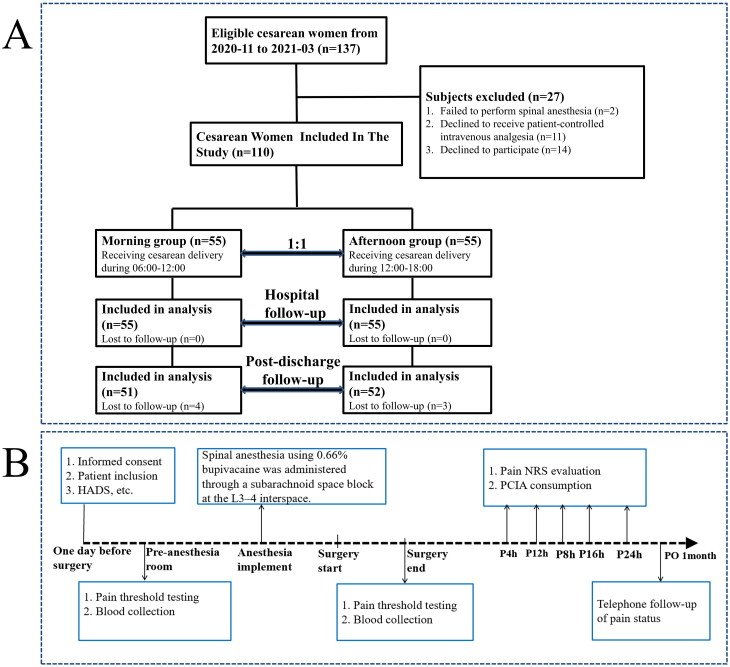
Flow diagram of patient inclusion (A) and study progress (B) through the prospective study. HADS: Hospital Anxiety and Depression Scale; NRS: Number Rating Scale; PCIA: patient-controlled intravenous analgesia.

For the prospective study (Figure 2A), from November 2020 to March 2021, women aged 20–40 years who underwent CS via a transverse incision under spinal anesthesia were grouped into the morning (surgery start time between 06:00 and 12:00, and the anticipative ending time was before 12:00) and afternoon groups (surgery start time between 12:00 and 18:00, and the anticipative ending time was before 18:00), as well as with ASA class I–II, singleton pregnancy, and voluntary receipt PCIA, were included. The exclusion criteria were women with chronic pain and using anti-inflammatory drugs or opioids within 3 months prior to the study, as well as being allergic to opioid analgesics and unable to cooperate during the study for any reason.

### Anesthetic and analgesic techniques

In the retrospective analysis, we screened women who underwent CS by spinal anesthesia, using 0.66% bupivacaine was administered through a subarachnoid space block at the L3–4 or L2–3 interspace and received PCIA with 2 ug/kg sufentanil and 12 mg ondansetron diluted in 100 mL of saline. The pump was programmed to have a load dose infusion of 2 ml, a background infusion rate of 2.0 mL/h, a PCIA dose of 2 mL, and a lockout period of 15 min. When necessary, nurses or doctors on duty provided additional analgesia as soon as possible and recorded it in the electronic medical system of the hospital.

The study procedure of the prospective study is shown in Figure 2B. Standardized spinal anesthesia using 0.66% bupivacaine was administered through a subarachnoid space block at the L3–4 interspace by an experienced anesthetist. All CS procedures in the prospective study were performed by the same surgical team using a standardized technique. PCIA was initiated immediately after the operation using a controlled infusion pump that delivered 2 ug/kg sufentanil and 12 mg ondansetron diluted in 100 mL of saline. The pump was programmed to have a load dose infusion of 2 ml, a background infusion rate of 2.0 mL/h, a PCIA dose of 2 mL, and a lockout period of 15 min. When the numerical rating scale (NRS) scores was ≥4, an extra analgesic (the non-opioids drugs, flurbiprofen) was administered. When the numerical rating scale (NRS) scores was ≥6, an extra analgesic (single dose of 0.1 ug/kg sufentanil diluted to 5 mL) was administered.

#### Outcome measures

In the retrospective study, we analyzed the frequency of analgesia requirement at 0–8, 8–16, and 16–24 h postoperatively, and the total frequency was calculated as the primary outcome. In the prospective study, the investigators continuously monitored postoperative pain intensity. Pain intensity at rest and during movement 24 h after CS was determined using the pain NRS (0–10; 0 was defined as no pain and 10 as intolerable pain). Pain ratings at rest and during movement and analgesic consumption were recorded by an investigator at 0, 4, 8, 12, 16, and 24 h postoperatively. In this study, the maximum NRS score for pain during the 24-h follow-up period was considered the primary outcome. The area under the curve (AUC) for the pain NRS during the first 24 h after CS was also calculated. In addition, the pain status 1 month postoperatively was also evaluated through telephone interviews.

In addition to subjective pain scores, we measured mechanical PPT and PTO both pre- and postoperatively. The mechanical PPT can be used to quantify pain perception in humans, while PTO can be used to quantify pain tolerance [20]. The experimental pain sensitivity was determined based on the results of pressure pain threshold (PPT) and pressure pain tolerance (PTO) before and after CS. The PPT was measured using a hand-held electronic mechanical algometer (YISIDA-DS2; Hong Kong, China) with a 0.1-cm^2^ probe, as reported in a previous study [20]. The lateral brachioradialis of the elbow joint on the dominant forearm was selected as the test location, and adjacent measurement points on the skin were identified using marks (X) as in a previous study [21]. The probe was positioned perpendicularly to the measurement point, and the investigator applied continuous pressure at approximately the same rate (0.3 kg/s) with the pre-established maximum force of 5 kg. Participants were asked to report ‘pain’ when they started to feel pain (PPT) while report ‘ok’ when they felt unbearable (PTO) during the stimulation, and the investigator applied the algometer to each of the two measurement points in a 60-s interval. The average values of the two measurements were calculated and recorded as the PPT and PTO.

Blood specimens (3–5 mL) were collected from the radial artery using a vacuum tube with heparin before and after CS. Serum was separated and stored at −80 °C, following which levels of inflammatory factors including interleukin-6 (IL-6) as well as endogenous opioid peptides including endorphin, enkephalin, and dynorphin were detected using an enzyme-linked immunosorbent assay (ELISA) kit.

#### Sample size determination

The retrospective cohort analysis was designed as an exploratory study. The propensity score-matched analysis was considered the primary comparison, and the frequency of extra analgesia requirements was considered the primary outcome. The power calculation of the propensity score-matched analysis indicated that a sample size of 104 was required in each group to detect a difference (4.5 ± 4.0 vs. 2.8 ± 2.5) in the frequency of extra analgesia requirements between the afternoon and morning groups with 95.5% power. This indicated that the sample size of the current retrospective study was sufficient.

The primary objective of the prospective study was to compare postoperative pain intensity between the morning and afternoon CS groups (the morning group: surgery start time between 06:00 and 12:00, and the anticipative ending time was before 12:00, and afternoon groups: surgery start time between 12:00 and 18:00, and the anticipative ending time was before 18:00). In our pilot analysis of women undergoing CS in the morning in our hospital, the maximum pain NRS score during the first 24 h post-CS was 3.7 ± 1.6. We assumed that the pain NRS score may increase by 25% when the surgery was performed in the afternoon. Based on a significance level of 0.05 and a power of 0.8, the minimum sample size required was determined to be 51 in each group (PASS, version 11.0; NCSS, Kayesville, UT). Considering the loss to follow-up in 5% of cases, we aimed to include 110 participants (55 in each group).

#### Statistical analysis

Data were analyzed using SPSS 19.0 and R statistical software. Statistical significance was defined as a two-sided *p*-value <0.05. Continuous data are presented as the mean ± standard deviation, while discontinuous data are presented as numbers (percentage). In the retrospective analysis, independent-samples *t*-tests were performed to examine differences in baseline data between the morning and afternoon groups. The chi-square test was used to compare CS history between the two groups. Mann–Whitney *U*-tests were used to compare the frequency of additional analgesia requirements between the two groups. Given the potential differences in baseline data between the morning and afternoon groups, a propensity score matching (PSM) analysis was performed. The propensity score was calculated based on age, height, weight, body mass index, gestational weeks, duration of surgery, and blood loss. Matching was performed using the 1:1 nearest-neighbor method without replacement under a logit model, which yielded 104 women in each group. Baseline data were also compared between the groups in the non-matched cohort.

For the prospective study, independent-samples *t*-tests, Mann–Whitney *U*-tests, or chi-square tests were used to analyze baseline and intraoperative data. NRS scores for pain at rest and during movement at different time points, as well as AUC values for pain NRS and PCIA consumption during the first 24 h after CS were compared between the groups using independent-samples *t*-tests or Mann–Whitney *U*-tests. Experimental pain sensitivity, including PPT and PTO, before and after CS were compared using repeated measures analyses of variance. Given large variations in serum inflammatory factors such as IL-6 and endogenous opioid peptides (endorphin, enkephalin, and dynorphin) among participants, changes in levels of these detected parameters were assessed using normalized values, which were calculated as the preoperative value divided by the postoperative value. The normalized values were then compared between the morning and afternoon groups.

## Results

The results of the retrospective study are shown in Figure 1A. A total of 636 women were screened for eligibility between September 2019 to February 2020, after the exclusion of 346 women according to the exclusion protocol criteria, 290 women were finally included, removal of 12 women (because of data missing), a total of 278 women undergoing CS (140 and 138 in the morning and afternoon groups, respectively) were included in the primary analysis (Figure 1A). The baseline demographic and clinical characteristics of the retrospective cohort are listed in Table 1. Before PSM, there were no significant differences in baseline data except for the surgery duration. The frequency of extra analgesia requirements in 8–16 h (1.1 ± 1.2 vs. 0.8 ± 1.1, *p* = 0.010) and the total frequency during the first 24 h after CS (4.5 ± 3.8 vs. 3.4 ± 2.9, *p =* 0.009) were significantly greater in the afternoon group than in the morning group (Figure 1B). After PSM, 104 women were included in each group, no significant differences in baseline data were observed. The frequency of extra analgesia requirements at 0–8 h (2.7 ± 2.7 vs. 1.8 ± 1.9, *p =* 0.021), 8–16 h (1.1 ± 1.1 vs. 0.6 ± 1.0, *p =* 0.002), and the total frequency during the first 24 h after CS (4.5 ± 4.0 vs. 2.8 ± 2.5, *p =* 0.002) were significantly higher in the afternoon group than in the morning group (Figure 1C).

**Table 1.** Baseline demographic and clinical characteristics of women receiving cesarean section in the retrospective cohort from September 2019 to February 2020 at a teaching hospital in China.

	Unmatched cohort			Matched cohort		
	Morning group (*n* = 140)	Afternoon group (*n* = 138)	*p* Value	Morning group (*n* = 104)	Afternoon group (*n* = 104)	*p* Value
Age (year)	30.3 ± 3.9	29.5 ± 4.2	0.088	30.1 ± 4.0	30.2 ± 4.1	0.878
Height (cm)	159.7 ± 4.5	158.8 ± 5.8	0.172	159.0 ± 4.7	159.4 ± 4.4	0.575
Weight (kg)	67.8 ± 8.1	68.1 ± 8.9	0.730	67.7 ± 9.1	67.7 ± 7.9	>0.999
BMI (kg/m^2^)	26.6 ± 3.1	27.0 ± 3.7	0.263	26.7 ± 3.1	26.6 ± 2.9	0.833
Gestational age (week)	38.6 ± 1.3	38.7 ± 1.9	0.555	38.7 ± 2.0	38.6 ± 1.3	0.935
History of caesarean section, *n* (%)	53(37.9)	43(31.2)	0.240	40(34.5)	42(36.2)	0.891
Surgery duration (min)	46.3 ± 14.3	50.3 ± 13.3	0.016	49.1 ± 13.6	48.8 ± 14.2	0.869
Blood loss (mL)	315 ± 171	310 ± 101	0.762	313 ± 113	307 ± 120	0.678

BMI: body mass index.

**Figure 3.**
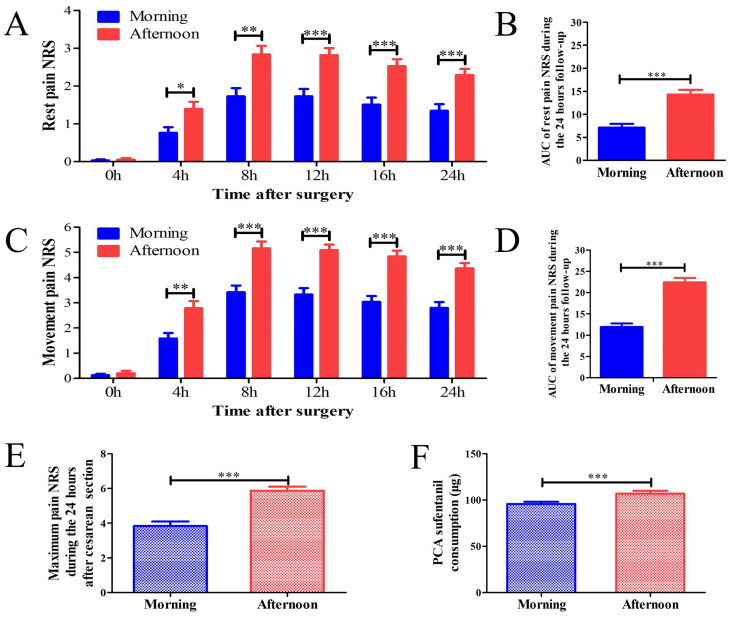
Comparisons of the outcomes of postoperative pain between the morning and afternoon groups. NRS scores for pain at rest (A) and during movement (C) at different time points, and the AUC values for pain NRS scores during the first 24 h after CS (B and D) are shown. The maximum pain NRS score during the first 24 h after CS (E) and sufentanil consumption (F) via PCIA in the morning and afternoon groups are shown. NRS: Number Rating Scale; AUC: area under the curve; PCIA: patient-controlled intravenous analgesia; **p < 0.01; **p < 0.001.

As shown in Figure 2A, in the prospective study, A total of 137 women undergoing CS were included (Figure 2), 27 women excluded according to the exclusion protocol criteria, 55 in each of the morning and afternoon groups were included in the final analysis. No significant differences were found between the groups in participants’ demographic characteristics or preoperative variables (Table 2). Between-group comparisons of NRS pain scores at rest and during movement are shown in Figure 3. At rest, pain intensity was significantly greater in the afternoon group than in the morning group at 4 (1.40 ± 1.38 vs. 0.76 ± 1.09, *p =* 0.015), 8 (2.84 ± 1.70 vs. 1.72 ± 1.61, *p =* 0.001), 12 (2.82 ± 1.38 vs. 1.73 ± 1.46, *p <* 0.001), 16 (2.53 ± 1.37 vs. 1.51 ± 1.37, *p <* 0.001), and 24 h (2.29 ± 1.21 vs. 1.35 ± 1.31, *p <* 0.001) post-CS (Figure 3A). During movement, pain intensity was also significantly greater in the afternoon group than in the morning group at 4 (2.78 ± 2.15 vs. 1.58 ± 1.63, *p =* 0.002), 8 (5.16 ± 2.01 vs. 3.42 ± 2.01, *p <* 0.001), 12 (5.09 ± 1.62 vs. 3.33 ± 1.89, *p <* 0.001), 16 (4.84 ± 1.71 vs. 3.03 ± 1.72, *p <* 0.001), and 24 h (4.36 ± 1.59 vs. 2.80 ± 1.67, *p <* 0.001) post-CS (Figure 3C). AUC values for NRS pain scores at rest (11.93 ± 5.92 vs. 7.11 ± 6.11, *p <* 0.001) and during movement (22.44 ± 7.48 vs. 14.29 ± 7.59, *p <* 0.001) 24 h after CS were significantly higher in the afternoon group than in the morning group (Figure 3B,D). The primary outcome of the study (i.e. maximum pain NRS) was also significantly higher in the afternoon group than in the morning group (5.9 ± 1.7 vs. 3.8 ± 1.9, *p <* 0.001; Figure 3E), as was total analgesia consumption (106.7 ± 22.3 vs. 95.7 ± 17.7 μg, *p <* 0.001; Figure 3F).

**Table 2.** Demographic characteristics, preoperative and intraoperative data in women receiving cesarean section of morning and afternoon groups in the prospective cohort from November 2020 to March 2021 at a teaching hospital in China.

Categorize the variables	Variables	Morning surgery (*n* = 55)	Afternoon surgery (*n* = 55)	*p* values
Demographic characteristics	Age (year)	31.3 ± 4.3	30.5 ± 3.8	0.295
	Height (cm)	158.4 ± 4.8	159.0 ± 4.4	0.447
	Weight (kg)	68.8 ± 6.8	69.5 ± 8.9	0.656
	BMI (kg/m^2^)	25.6 ± 1.5	25.3 ± 1.4	0.422
	Smoking, *n* (%)	0 (0.0)	3 (5.5)	0.242
	Drinking, *n* (%)	0 (0.0)	1 (1.8)	0.999
Reproductive history	History of caesarean section, *n* (%)	33 (60.0)	31 (56.4)	0.699
	Gestational diabetes mellitus, *n* (%)	13 (23.6)	8 (14.5)	0.225
	Other complication in pregnancy, *n* (%)	12 (21.8)	19 (34.5)	0.138
	Gestational age (week)	38.9 ± 1.1	38.7 ± 1.2	0.376
Clinical parameters	Systolic pressure	112.1 ± 20.0	112.0 ± 13.9	0.987
	Diastolic pressure	74.8 ± 13.4	74.4 ± 12.1	0.860
	Hepatic cholestasis, *n* (%)	4 (7.3)	2 (3.6)	0.675
Laboratory test	White blood cell count (10^9^/L)	7.3 ± 2.8	6.5 ± 3.3	0.136
	Neutrophil count (10^9^/L)	6.6 ± 2.3	6.8 ± 2.4	0.554
	Neutrophil lymphocyte ratio	4.8 ± 1.9	5.1 ± 2.6	0.473
Mental health assessment	HADS-anxiety	5.4 ± 3.2	5.2 ± 2.6	0.952
	HADS-anxiety ≥ 8, *n* (%)	10 (18.2)	13 (23.6)	0.482
	HADS-depression	3.5 ± 2.9	3.2 ± 2.3	0.831
	HADS-depression ≥ 8, *n* (%)	3 (5.5)	2 (3.6)	0.647
Intraoperative data	Surgery duration (min)	48.2 ± 18.4	48.8 ± 15.8	0.844
	Blood loss (mL)	270.9 ± 68.5	277.5 ± 82.0	0.656

BMI: body mass index; HADS: Hospital Anxiety and Depression Scale.

**Figure 4.**
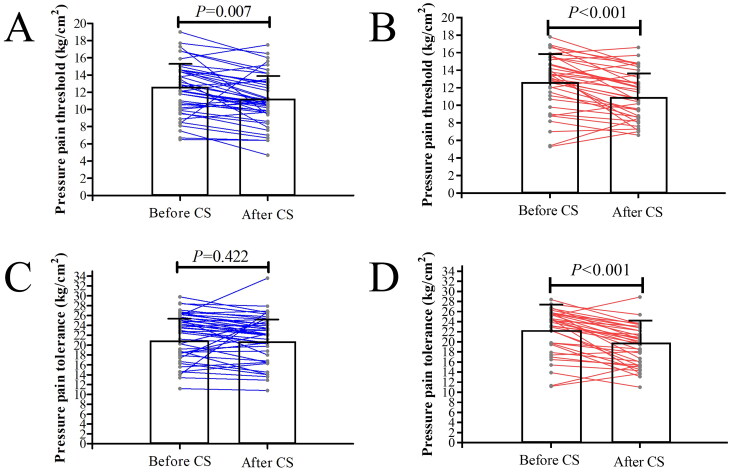
The pressure pain threshold (A and B) and pressure pain tolerance (C and D) before and after cesarean section (CS) in the morning and afternoon groups.

**Figure 5.**
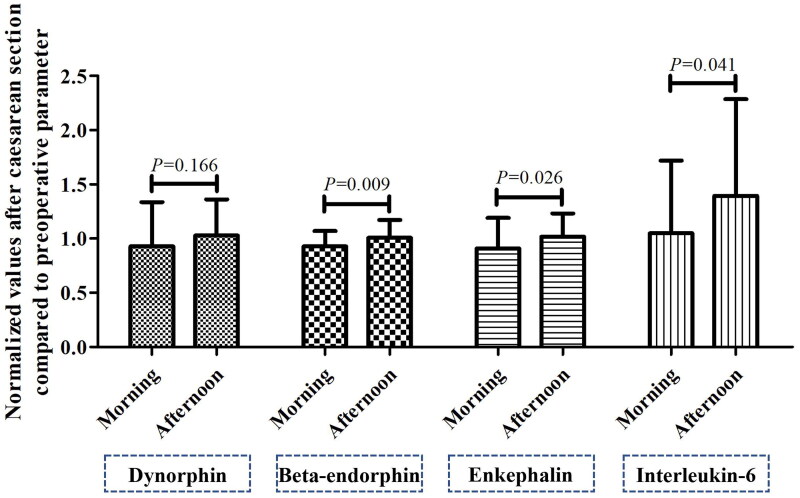
Changes in serum endorphin and interleukin-6 levels after cesarean section when compared with preoperative values in the morning and afternoon groups.

No significant between-group difference in PPT (12.4 ± 3.0 vs. 12.7 ± 3.2 kg/cm^2^, *p =* 0.624) or PTO (21.9 ± 4.7 vs. 22.1 ± 4.5 kg/cm^2^, *p =* 0.818) was observed before CS. Although there was no significant between-group difference (11.3 ± 3.2 vs. 11.1 ± 2.8 kg/cm^2^, *p =* 0.778) in PPT scores after CS, PTO scores were significantly higher in the morning group than in the afternoon group after CS (21.5 ± 4.7 vs. 19.1 ± 3.9 kg/cm^2^, *p =* 0.015). In addition, as shown in Figure 4, the PPT after CS was significantly lower than that before CS in both the morning (*p =* 0.007) and afternoon groups (*p <* 0.001; Figure 4). Conversely, PTO scores after CS were significantly lower than those obtained before CS in the afternoon group (*p <* 0.001), while no significant change was observed in the morning group (*p =* 0.442). There were no significant between-group differences in ELISA results preoperatively (Table 2). Following CS, there were no significant changes in any of the three types of serum endorphins in the afternoon group (*p* > 0.05; Figure 5). However, in the morning group, levels of all three types of serum endorphins after CS were significantly lower than those observed before CS (*p <* 0.05). The change in the level of IL-6 following CS was significantly greater in the afternoon group than in the morning group (*p =* 0.041).

The pain status 1 month after CS is shown in Table 3. No significant differences were observed in any of the investigated parameters. In total, 78.4% of women in the morning group and 82.7% in the afternoon group reported pain one month after CS, and the NRS score for pain was higher in the afternoon group than in the morning group (1.87 ± 1.27 vs. 1.49 ± 1.06, *p =* 0.056).

**Table 3.** Pain characteristics of women at one month after cesarean delivery in morning and afternoon groups in the prospective cohort from November 2020 to March 2021 at a teaching hospital in China.

	Morning surgery (*n* = 51)	Afternoon surgery (*n* = 52)	*p* Values
Complains of pain, *n* (%)	40(78.4)	43(82.7)	0.585
Pain NRS	1.49 ± 1.06	1.87 ± 1.27	0.056
Has sleep disturbance, *n* (%)	2(3.9)	5(9.6)	0.226
Has mood disturbance, *n* (%)	2(3.9)	3(5.8)	0.509
Analgesic use, *n* (%)	0(0.0)	0(0.0)	1.000

NRS: Number Rating Scale.

## Discussion

In this study, we aimed to determine whether the timing of cesarean delivery (morning vs. afternoon) influences postoperative pain intensity and analgesic requirements. We found that time of day had a notable impact on several pain-related outcomes. Women who underwent CS in the afternoon generally required more postoperative analgesia and experienced higher pain intensity in the first 24 h compared to those who had morning procedures. Afternoon CS was also associated with a greater reduction in postoperative pain tolerance (despite similar pain threshold changes as morning CS) and a more pronounced physiological stress response (evidenced by differences in β-endorphin and IL-6 levels). By one month after surgery, a majority of patients in both groups reported persistent pain, with no significant difference in long-term pain incidence between morning and afternoon cases.

In the retrospective study, the 278 eligible participants were divided into two groups based on whether CS was performed from 6:00–12:00 or from 12:00–18:00. The results showed that the frequency of extra analgesic demand during the first 24 h post-CS was significantly higher among those who underwent the procedure in the afternoon. Our PSM analysis also indicated that the frequency of postoperative analgesia requirement was greater in patients undergoing CS in the afternoon than in those undergoing CS in the morning, which yielded similar findings. However, because of the natural limitations of retrospective studies, it is difficult to control other potential factors that may affect perioperative analgesia outcomes. Our recent study also showed that the analgesic effect of opioids in the morning was stronger compared to that in the afternoon [22], which may contribute to the current finding. In addition, we did not systematically evaluate pain intensity or other indicators using objective methods, highlighting the need for further studies to examine the effect of daytime variation on pain after CS.

In the prospective study, our analysis indicated that the maximum pain level was significantly higher in the afternoon group than in the morning group. Results of AUC also indicated that the overall pain intensity after CS was significantly higher for procedures performed in the afternoon than for those performed in the morning. A previous animal study found that noxious stimulation at different times can cause diurnal variation in pain reactions [23], indicating the effect of time variation in nociceptive response. In a recent clinical study regarding patients receiving selective laparoscopic abdominal surgeries under general anesthesia, increased pain perception was reported when the operation was performed in the evening than in the morning [24]. Combined with these findings, it is speculated that pain intensity is associated with time points of surgical injury. In addition, the results showed that total postoperative sufentanil consumption was higher in the afternoon group than in the morning group. The current combination of retrospective and prospective data indicates that an one-size-fits-all strategy is not suitable for analgesic management after CS, and that postoperative pain may be undertreated among patients undergoing CS in the afternoon. Conversely, analgesic-related side effects should be considered to ensure that patients undergoing CS in the morning are not overtreated for postoperative pain. Consistent with our hypothesis, the need for additional analgesia was higher after afternoon surgeries. In the retrospective cohort, patients whose CS took place in the afternoon had a significantly greater frequency of extra analgesic requests in the first 24 h postoperatively compared to the morning group, and this was corroborated by higher opioid consumption via PCIA in the prospective cohort. One explanation is the circadian variation in pharmacodynamics; our observational study found that opioids like sufentanil produce stronger analgesic effects in the morning than in the afternoon [22], which could render afternoon patients less responsive to standard doses. Another consideration is potential differences in pain perception or care patterns later in the day, although our standardized protocols (same surgical team and analgesic regimen for both groups) aimed to minimize such variability. These findings highlight that an one-size-fits-all analgesic strategy may not be optimal—postoperative pain could be undertreated in afternoon patients under an uniform regimen, a novel consideration for tailored pain management after CS.

Our prospective data also demonstrated that afternoon surgeries led to higher pain scores throughout the first day, aligning with prior evidence of diurnal variation in pain sensitivity. The peak pain NRS and overall 24-hour pain burden (AUC) were significantly greater in the afternoon group than in the morning group. This result supports the concept that nociceptive responses can fluctuate with time of day: for example, experimental studies have shown more intense pain behaviors at certain times (corresponding to the active phase) than others [25]. Clinically, patients undergoing abdominal surgery in the evening experienced higher postoperative pain and worse sleep quality than those with morning operations [24]. Our study extends these observations to cesarean delivery under spinal anesthesia, suggesting that the circadian timing of surgical injury influences acute post-CS pain intensity. Notably, because all patients received identical anesthetic and analgesic protocols and surgeries were performed by the same team, factors such as surgical technique or anesthetic management are unlikely to explain the pain gap. This finding provides the first clinical evidence in CS patients that chronobiological factors inherent to the patient (e.g. hormonal or receptor sensitivity fluctuations) may modulate postoperative pain perception.

In the current study, postoperative PPT decreased significantly when compared with preoperative values in both the morning and afternoon groups. Mechanical pain threshold has been used to evaluate postoperative hyperalgesia in many previous studies, and it has been demonstrated that the degree of PPT reduction is positively correlated with hyperalgesia [26,27]. Therefore, the PPT reductions observed in the current study indicate that post-CS hyperalgesia in the morning group was similar to that in the afternoon group. However, there were no differences between pre- and postoperative PTO in the morning group, although postoperative PTO was significantly lower than the preoperative value in the afternoon group. It is believed that suprathreshold noxious stimulation better predicts clinical pain and analgesia requirements than pain thresholds [28]. Moreover, many studies focusing on the prediction of postoperative pain have shown that PTO is a better predictor [11,29]. We speculate that the significant drop in pain tolerance observed after afternoon surgeries – not seen in morning surgeries – may be a key factor underlying the more severe pain reported by the afternoon group. In other words, patients who underwent CS in the afternoon appeared to have an increased sensitivity to pain above their threshold (lower tolerance), which could have manifested as higher pain scores and greater analgesic needs.

In the current study, endogenous opioid levels (especially those of β-endorphin) significantly decreased following CS in the morning group. Because the release of endogenous opioid peptides from the pituitary gland into the circulation is associated with the systemic stress response [30], our results indicate that stress levels may have decreased after spinal anesthesia in the morning group. This is consistent with the results of several previous studies showing that the expression of β-endorphin in the peripheral blood of pregnant women significantly decreases after receiving spinal anesthesia, which has been shown to be associated with reduced stress responses [31,32]. On the contrary, there was no significant change in the afternoon group, indicating that stress responses may have been more pronounced in the afternoon group than in the morning group despite receipt of the same surgical procedure and anesthesia protocol. In addition, the change in the serum IL-6 level was significantly greater in the afternoon group than in the morning group. Increased postoperative IL-6 expression has been shown to positively correlate with postoperative pain [33–35]. Thus, we speculated that higher postoperative pain levels in the afternoon group may have been associated with a higher level of CS-induced stress.

In this study, we also examined pain levels 1 month after CS via telephone interviews to explore the influence of different surgery times on long-term postoperative pain. The results showed that most participants in both groups still experienced pain. A study on the development of pain after CS reported that 81% of the pain disappeared or improved 22 ± 9 days post-CS, with 8% of the participants being primigravid [36]. Our recent study of Chinese women also reported that the proportion of primiparas who still experienced pain 1 month postoperatively reached 62.2%, with 48.2% of the participants being primigravid [37]. Previous studies have confirmed that poor control of acute postoperative pain is a high-risk factor for persistent pain after CS [38]. Although there was no significant between-group difference in the incidence of pain 1 month postoperatively in this study (*p =* 0.056), the higher average pain score 1 month postoperatively in the afternoon group may have been related to poor postoperative acute pain control. These findings, together with our results, highlight that persistent post-cesarean pain is not uncommon and merits attention. It is well established that poor control of acute postoperative pain is a major risk factor for developing persistent pain after CS. Although our study was not powered to detect differences in chronic pain, the slightly higher long-term pain in the afternoon group may be related to their worse acute pain in the immediate postoperative period. This underscores the importance of adequately managing acute pain, especially in patients undergoing afternoon surgeries, to potentially reduce the risk of chronic post-surgical pain.

Some strengths and limitations of this study should be noted. A key strength is the two-phase design, wherein a retrospective analysis provided initial insight that was then validated by a prospective controlled study. This approach enhances confidence in the findings. We also employed objective pain measurements (PPT, PTO) and biomarker analysis to complement subjective pain scores, providing a more comprehensive assessment of pain and its physiological correlates. However, first, considering that most surgeries were performed during the daytime, we only included women who underwent elective CS from 6:00 to 18:00. Thus, our findings cannot be directly extrapolated to nighttime or emergency CS cases when surgical timing is unplanned. Second, unified PCIA was adopted for postoperative analgesia in this study, and no other analgesic methods were included, such as continuous spinal analgesia, so our results specifically pertain to intravenous opioid-based analgesia. It remains to be determined whether the observed time-of-day effect on pain would also be seen under different analgesic techniques. Third, in this study, only endorphin and IL-6 were detected. The potential role of other mediators, such as serum progesterone, in the difference of pain after CS needs to be further explored. In addition, in this study, we did not measure the body temperature of the patients, which may reflect the rhythm information of the patients. Furthermore, in this study, we primarily focused on wound pain but did not evaluate and record the pain from the urinary catheter. These contents should be explored in future studies. Finally, due to the relatively lower incidence of postoperative chronic pain and the small sample size of the study, postoperative follow-up was conducted for only 1 month, highlighting the need for larger studies to examine the effects of time variation on chronic pain after CS.

One important potential difference between morning and afternoon surgeries is the effect of provider fatigue or a busier operative schedule in the afternoon, which could conceivably impact patient pain outcomes. We addressed this concern in our study design and analysis. All CS cases in the prospective study were performed by the same experienced surgical team following a standardized procedure, so variability in surgical technique or speed was minimized. Moreover, our comparison of baseline intraoperative data showed no significant differences in duration of surgery or blood loss between the morning and afternoon groups, suggesting that surgical complexity was comparable. Thus, we believe that surgeon fatigue or case load did not substantially influence the difference in pain outcomes between morning and afternoon groups in our study. Additionally, in our hospital, surgical patients (including those for afternoon cases) are typically admitted and prepared by outpatient staff earlier in the day, so the burden on the surgical team is not necessarily greater in the afternoon. For these reasons, it is unlikely that differences in provider workload explain the higher pain observed after afternoon CS.

In summary, the current results indicate that CS performed in the afternoon may induce greater postoperative pain and a greater requirement for postoperative analgesia than CS performed in the morning. This phenomenon may be associated with lower pain tolerance and a more severe postoperative stress response. These findings suggest that temporal (circadian) factors should be taken into account when developing personalized, targeted analgesia strategies for patients undergoing CS. Moreover, the timing of surgery might affect postoperative pain outcomes in other types of surgery as well, an area that deserves further exploration. Optimizing the timing of elective surgeries or adjusting pain management protocols according to time of day could be considered in the future to improve postoperative comfort and outcomes.

## Data Availability

The data that support the findings of this study are available from the corresponding author, GD, upon reasonable request.
